# Inhibitory Effect of NMDA Receptors in the Ventral Tegmental Area on Hormonal and Eating Behavior Responses to Stress in Rats

**DOI:** 10.1155/2014/294149

**Published:** 2014-08-07

**Authors:** Zohreh Sadat Nasihatkon, Maryam Khosravi, Zahra Bourbour, Hedayat Sahraei, Mina Ranjbaran, Seyedeh Maryam Hassantash, Mohammad Sahraei, Kefayat Baghlani

**Affiliations:** ^1^Department of Biology, Faculty of Biological Science, North Tehran Branch, Islamic Azad University, Tehran 19395-6558, Iran; ^2^Neuroscience Research Center, Baqiyatallah (a.s.) University of Medical Sciences, Tehran 19395-6558, Iran; ^3^Faculty of Dentistry, Shahid Beheshti University of Medical Sciences, Tehran 19395-6558, Iran; ^4^Department of Physiology and Biophysics, Faculty of Medicine, Baqiyatallah (a.s.) University of Medical Sciences, Tehran 19395-6558, Iran

## Abstract

*Background*. Stress and its consequences are among the causes of accidents.* Objective*. The effects of intraventral tegmental area (I-VTA) memantine on the plasma corticosterone and eating parameters disturbance induced by acute stress were investigated.* Methods*. Male Wistar rats (W: 250–300 g) were divided into control and experiential groups, each of which received memantine either intra-VTA or peripherally. One week after bilateral cannulation, the rats received memantine (1 and 5 *μ*g/Rat) five min before electroshock stress. The other experimental groups received memantine (1 and 5 mg/kg) intraperitoneally 30 min before stress. The control groups received saline or memantine but did not experience stress. Food and water intake and plasma corticosterone level were recorded.* Results*. Results showed that stress decreases food intake but does not change water intake and increase in plasma corticosterone level. Intraperitoneal memantine administration slightly inhibits the stress effects on food intake. However, water intake and plasma corticosterone level were increased. Intra-VTA memantine reduces the effects of stress on corticosterone and water intake.* Conclusion*. It could be concluded that inhibition of glutamate NMDA receptors in the VTA by memantine leads to the inhibition of the eating behavior parameters and plasma corticosterone level disturbance induced by stress in rats.

## 1. Introduction

It is now clear that exposure to stressful events elicit sever consequences in the veterans; despite intense investigations, the exact mechanisms underlying these effects are not fully understood [1]. The ventral tegmental area (VTA) of the midbrain contains the cell bodies of the mesolimbic dopamine system which serves as a target for molecules released during stress [2–4]. A considerable body of research also indicates that glutamatergic neurotransmission within the ventral tegmental area might be a critical neurochemical determinants of stress effects [5–9]. Glutamate induces its effects in part by activation of its N-methyl-D-aspartate (NMDA) receptors, which are extensive distribution in the VTA [9, 10]. Activation of NMDA receptors stimulates the entry of calcium ions into the cells and thus increases the activity of dopamine neurons and also the stimulation of dopamine secretion in the target areas of these neurons, which were described above [11–13]. Glutamatergic projections from the medial prefrontal cortex are the main glutamate sources to the VTA and it seems that these inputs along with the dopaminergic projections from ventral tegmental to nucleus accumbens, amygdala, and prefrontal cortex modulate the activity of stress system in the animals [6, 7, 9, 10, 14–21].

Various investigations indicate that glutamate system may have a role in modulating stress side effects. In this regard, studies indicated that inhibition of NMDA receptors decreases stress side effects [22, 23]. These studies indicated that peripheral injection of memantine in the rats subjected to chronic mild stress for seven days inhibit stress-induced anorexia, adrenal gland weight excess, and plasma corticosterone elevation. The drug also decreases BDNF levels elevation induced by stress in the prefrontal area of cerebral cortex [22, 23]. On the other hand, it has been found that memantine injection prevents stress-induced cell production decline in the hippocampus. In this regard, restraint stress applied for 2 h/day for seven days reduces cell proliferation in hippocampus which was inhibited by memantine [24].

Moreover, memantine may also be useful in treatment of posttraumatic stress disorder (PTSD) both in animal models and human [25–27]. Considering the conducted researches, in the present study, the fact that the effect of memantine injection either peripherally or into VTA on plasma corticosterone levels and some eating behavior parameters such as food and water intake and delay to eating changes after acute stress in male Wistar rats was investigated.

## 2. Materials and Methods

### 2.1. Animals

Wistar rats (W: 250–300 g) obtained from Iran Pasteur Institute were used in this study (4 rat/cage). The animals were not fasted before the test and used standard food and water. The animals were kept in their home cages for one week before the experiments and their food and water intake were measured as the base of the food and water intake. Dark/light period of 12/12 h and humidity of 50% were conducted for the animals. All the experiments were conducted according to Animal Rights Guidelines, Deputy of Research, Baqiyatallah University of Medical Sciences. In each series of experiments, 8 animals were used.

### 2.2. Drugs

Memantine hydrobromide (Sigma, USA), ketamine hydrochloride, and diazepam (Sigma, USA) were used in this study. All drugs were dissolved in sterile saline and injected i.p. at the dose of 1 mL/kg. For I-VTA injections, memantine was dissolved in the sterile saline and injected to the animals in volume of 0.25 *μ*L/Rat. Since ketamine hydrochloride is a NMDA receptor antagonist, after the surgical processes, each animal was allowed one week for recovery from the ketamine and surgery.

### 2.3. Stress Induction

Stress box was used to perform this experiment. This device has 9 compartments composed of Plexiglas. The floor of the compartments had steel wires and was connected to the electroshock device, which was controlled by a PC. The severity and duration of electroshock induction were 60 mV, 10 Hz, and for 100 Sec [28]. Animals were placed in the apparatus 30 min before the stress beginning and maintained in the apparatus after stress termination for additional 30 min. In experimental groups, the animals received 1 and 5 mg/kg intraperitoneally and/or 1 and 3 *μ*g/rat intra-VTA memantine 5 min before stress induction. Blood samples were taken after stress termination in order to measure the plasma corticosterone concentration as stress index in these animals.

Animals of the control groups were treated similar to the ones in the case group, but they received saline instead of memantine. After the termination of stress, the animals were returned to their cage and time interval between their return and the beginning of feeding was measured and declared as delay to eating index. Moreover, food intake of each animal was measured as another behavioral indicator. Blood samples were taken from retroorbital sinus of the rats before or after stress termination.

### 2.4. Surgical Procedures

To inject memantine into VTA, the animals were first anesthetized by intraperitoneal injection of ketamine (50–75 mg/kg) and diazepam (5–7 mg/kg). Two stainless steel guide cannulas (Gauge 21) were bilaterally placed within the VTA. These cannulas were fixed using two screws and dental acrylic. Coordinates for cannulas were as follows: AP: −4.8 mm from Bregma, L: 1 mm from central line, and V: 7.5 mm from the surface of skull [29]. Injection into this area was performed by a stainless steel injection cannula number 30 which was attached to a 5 *μ*L Hamilton syringe by a plastic cannula (injection volume was 0.25 mL on each side). After the tests termination the animals were deeply anesthetized b ketamine (100 mg/kg) and their brains were fixed using Trans cardiac fixation method. The site of injection was specified by a specialist.

### 2.5. Histology

**Figure 1:**
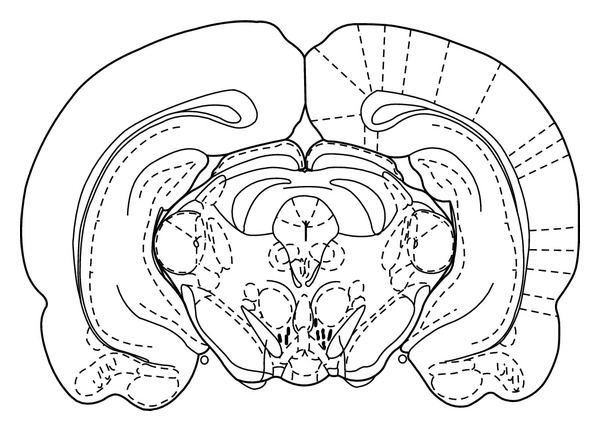
Location of cannula tips in the ventral tegmental area of animals used in the study. Symbol (∣) indicates where the cannula tips were placed in the ventral tegmental area.

After the completion of testing, all animals were anesthetized and received a transcardiac perfusion with 0.9% normal saline followed by 10% buffered formalin. The brains were removed, blocked, and cut coronally in 40 *μ*m sections through the cannula placements. The tissues were stained with cresyl violet and were examined by light microscopy by an unfamiliar observer to the behavioral data. Only the animals with correct cannula placements were included in the data analysis (Figure 1).

### 2.6. Statistical Analysis

Data were expressed as Mean ± SEM. In order to analyze the data, two-way analysis of variance (two-way ANOVA) was applied for the analysis of the differences between memantine treated groups using memantine and stress as factors. When two-way analyses showed a significant difference, the Tukey post hoc were used. *P* < 0.05 was considered significant.

## 3. Results

### 3.1. Effects of Peripheral and/or I-VTA Memantine on Plasma Corticosterone Level after Stress

**Figure 2:**
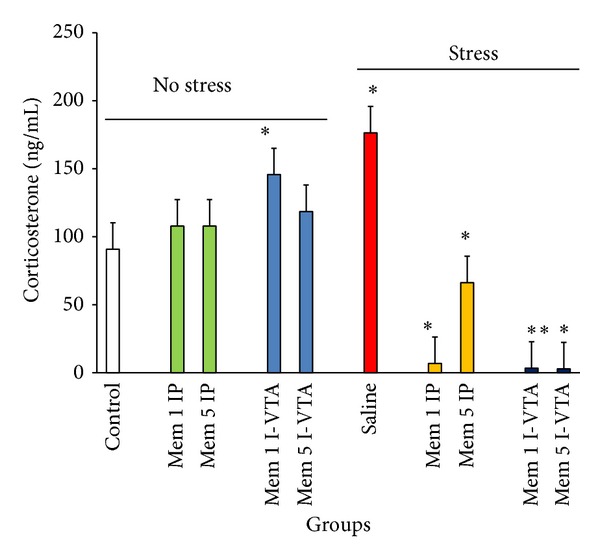
The effects of stress, memantine, and combination of stress and memantine on the plasma corticosterone level in rats. The animals received different doses of memantine either intraperitoneally or intra-VTA before each stress session. The control groups received sterile saline. Each point is mean ± SEM for 8 animals. *P < 0.05 and **P < 0.01 different from their respective controls.

In this series of experiments, 12 groups of animals (*n* = 7/group) were used. Six groups of them underwent surgery and cannulation as mentioned in the Materials and Methods section; after 7 days of recovery, they were exposed to electrical foot shock. Test groups received bilateral different doses of memantine (1 and 5 *μ*g/rat) or saline (in the same volume) within their VTA (0.25 *μ*L/rat/side) 5 min before stress induction. The control groups received the same dosage of memantine or saline without stress exposure. Other six groups received memantine (1 and 5 mg/kg) or saline (1 mL/kg) intraperitoneally with or without stress exposure. The results indicated that stress dramatically increases plasma corticosterone level and memantine administration reduces the stress effects both when injected as IP or I-VTA (two-way ANOVA within group comparison: memantine effect: F(9, 63) = 3.21, *P* < 0.01; stress effect: F(1, 63) = 2.79, *P* < 0.05; memantine × Stress effects: F(9, 63) = 4.36, *P* < 0.01) (Figure 2).

### 3.2. Effect of Peripheral or I-VTA Bilateral Memantine Administration on Food and Water Intake after Stress

**Figure 3:**
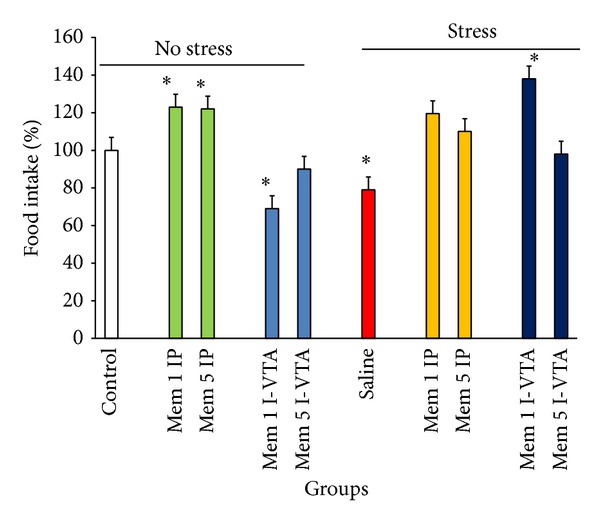
The effects of stress, memantine, and combination of stress and memantine on food intake in rats. The animals received different doses of memantine either intraperitoneally or intra-VTA before each stress session. The control groups received sterile saline. Each point is mean ± SEM for 8 animals. *P < 0.05 different from their respective controls.

**Figure 4:**
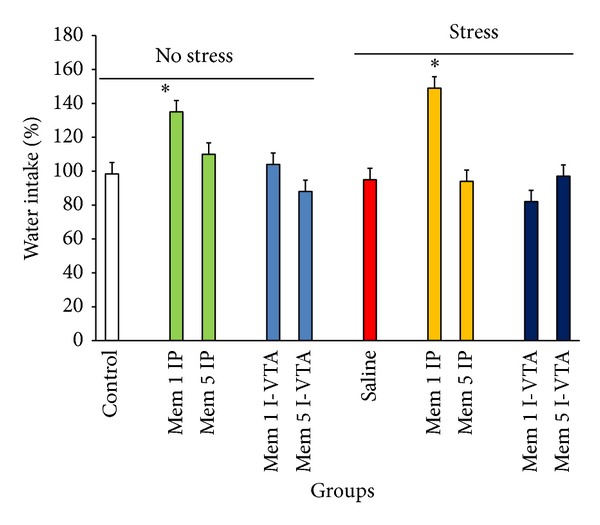
The effects of stress, memantine, and combination of stress and memantine on water intake in rats. The animals received different doses of memantine either intraperitoneally or intra-VTA before each stress session. The control groups received sterile saline. Each point is mean ± SEM for 8 animals. *P < 0.05 different from their respective controls.

Our data indicated that memantine by itself increases or decreases food and water intake when injected peripherally or I-VTA, respectively (Figures 3 and 4). However, stress decreased food intake and did not change water intake in the animals (Figures 3 and 4). Treatment with memantine increased food intake under both nonstress and stress conditions (two-way ANOVA within group comparison: memantine effect: F(9, 63) = 4.18, *P* < 0.01; stress effect: F(1, 63) = 5.68, *P* < 0.01; Memantine × Stress effects: F(9, 63) = 5.09, *P* < 0.01) (Figures 3 and 4).

### 3.3. Effect of Peripheral or I-VTA Memantine Administration on Delay to Eating Induced by Stress

**Figure 5:**
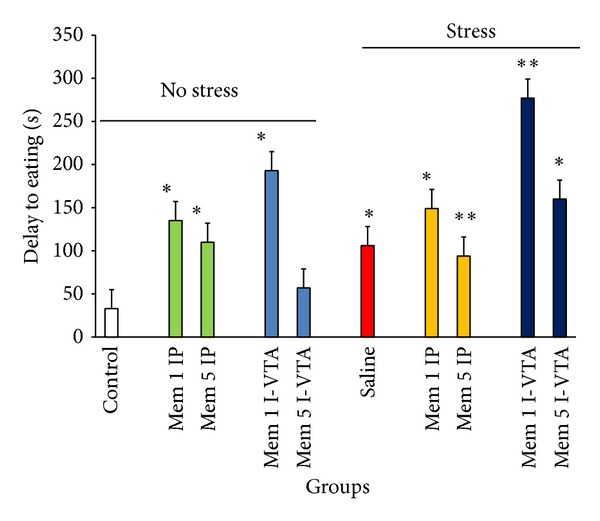
The effects of stress, memantine, and combination of stress and memantine on delay to eating in rats. The animals received different doses of memantine either intraperitoneally or intra-VTA before each stress session. The control groups received sterile saline. Each point is mean ± SEM for 8 animals. *P < 0.05 and **P < 0.01 different from their respective controls.

In this series of the experiments, the above-mentioned groups were returned to their cages 30 min after the termination of stress and their delay to eating was measured. The results indicated that in the negative control group (which were placed in the stress box but not exposed to stress), delay to eating time was very short after the animals were returned to their home cages (Figure 5). This time was dramatically increased for control stress group (Figure 5). However, the time to initiating the eating in the animals which received memantine (either IP or I-VTA) was increased significantly (two-way ANOVA within group comparison: memantine effect: F(9, 63) = 4.79, *P* < 0.01; stress effect: F(1, 63) = 3.19, *P* < 0.01; Memantine × Stress effects: F(9, 63) = 6.3, *P* < 0.01) (Figure 5).

## 4. Discussion

The present study was designed to respond to the question whether a modulation of NMDA glutamate receptors within the VTA ameliorates the plasma corticosterone levels and some eating behavior parameters changes induced by an uncontrollable stress or not. The findings however have shown that inhibition of these receptors inhibit the stress effects and promote the overall animal status.

Several studies have shown that stress induces several hormonal responses from which the corticosterone increment was considered as the main response [1]. Our data indicated that corticosterone plasma level was increased in the stressed animals. In agreement with our finding there are data indicating that different kinds of stress including the method which is used in the present study increase corticosterone plasma level in both human [30] and rats [31]. Plasma corticosterone increment is postulated to be the result of hypothalamus-pituitary-adrenal axis activity [1]. Surprisingly, the plasma corticosterone level in the animals which received memantine also increased significantly. In agreement with our finding, Réus et al., 2012, have shown that memantine in rats increases plasma corticosterone slightly [23]. However, the researchers used the dose of 20 mg/kg of the drug, whereas the dose of 10 mg/kg was ED 50% in our study. One possible reason for the different data may be due to different type of animals used in these two studies [23]. It is important to be mentioned that in some cases the lower dose of memantine was more effective.

The exact mechanism(s?) by which memantine induces corticosterone release is not understood. It must be noted that memantine increases plasma corticosterone and adrenocorticotropine hormone (ACTH) levels in the rats [32]. Our data indicated that at least one of the brain sites for memantine effects on corticosterone may be the ventral tegmentum area. However, the precise neuroanatomical relationship between VTA and the adrenal gland is not understood and remains to be examined. Our data also indicated that memantine affects the brain reward system and also manipulation of brain reward system interacts with stress system, the fact that was mentioned in several studies [8]. Studies have indicated that glutamate NMDA receptors are located on the cell membrane in the adrenal cortex and inhibition of these receptors may increases the corticosteroids release from these cells [33]. Similar mechanism may be involved in the results obtained in the present study for memantine. However, it is not understood why administration of memantine inhibits stress induced corticosterone release. One possible mechanism is that memantine may inhibit central glutamatergic mechanisms involved in the mediation of stress-induced CRF release. This hypothesis is supported by the fact that all of the stress side effects which are related to the CRF including food intake inhibition and anorexia also were inhibited by memantine pretreatment in the present study.

Memantine treatment either intraperitoneally or intra-VTA in both stressed and nonstressed groups in different doses increased delay to eating time in the animals. It is an interesting finding and there is no data for discussion in this regard. However, it is clear that memantine even potentiates the stress effect in this regard. It is not clear from our finding how the drug can induce such responses but it may function between different parts of the brain which are involved in response to stress with memantine.

As a general conclusion, it seems that intra-VTA and peripheral administration of antagonists of NMDA glutamate receptors (memantine) could decrease plasma corticosterone levels elevation as well as eating behavior parameters such as food and delay to eating changes caused by foot electrical shock stress in animals; this effect could be due to the central and also peripheral effects of memantine.
